# Experience with clinical cerebral autoregulation testing in children hospitalized with traumatic brain injury: Translating research to bedside

**DOI:** 10.3389/fped.2022.1072851

**Published:** 2023-01-10

**Authors:** Thitikan Kunapaisal, Anne Moore, Marie A. Theard, Mary A. King, Randall M. Chesnut, Monica S. Vavilala, Abhijit V. Lele

**Affiliations:** ^1^ Department of Anesthesiology and Pain Medicine, University of Washington, Seattle, WA, United States; ^2^ Harborview Injury Prevention, and Research Center, University of Washington, Seattle, WA, United States; ^3^ Cerebrovascular Laboratory, Harborview Medical Center, Seattle, WA, United States; ^4^ Department of Pediatrics, University of Washington, Seattle, WA, United States; ^5^ Department of Neurological Surgery, Harborview Medical Center, University of Washington, Seattle, WA, United States

**Keywords:** transcranial Doppler, children, traumatic brain injury, safety, clinical practice, feasibility

## Abstract

**Objective:**

To report our institutional experience with implementing a clinical cerebral autoregulation testing order set with protocol in children hospitalized with traumatic brain injury (TBI).

**Methods:**

After IRB approval, we examined clinical use, patient characteristics, feasibility, and safety of cerebral autoregulation testing in children aged <18 years between 2014 and 2021. A clinical order set with a protocol for cerebral autoregulation testing was introduced in 2018.

**Results:**

25 (24 severe TBI and 1 mild TBI) children, median age 13 years [IQR 4.5; 15] and median admission GCS 3[IQR 3; 3.5]) underwent 61 cerebral autoregulation tests during the first 16 days after admission [IQR1.5; 7; range 0–16]. Testing was more common after implementation of the order set (*n* = 16, 64% after the order set vs. *n* = 9, 36% before the order set) and initiated during the first 2 days. During testing, patients were mechanically ventilated (*n* = 60, 98.4%), had invasive arterial blood pressure monitoring (*n* = 60, 98.4%), had intracranial pressure monitoring (*n* = 56, 90.3%), brain-tissue oxygenation monitoring (*n* = 56, 90.3%), and external ventricular drain (*n* = 13, 25.5%). Most patients received sedation and analgesia for intracranial pressure control (*n* = 52; 83.8%) and vasoactive support (*n* = 55, 90.2%) during testing. Cerebral autoregulation testing was completed in 82% (*n* = 50 tests); 11 tests were not completed [high intracranial pressure (*n* = 5), high blood pressure (*n* = 2), bradycardia (*n* = 2), low cerebral perfusion pressure (*n* = 1), or intolerance to blood pressure cuff inflation (*n* = 1)]. Impaired cerebral autoregulation on first assessment resulted in repeat testing (80% impaired vs. 23% intact, RR 2.93, 95% CI 1.06:8.08, *p *= 0.03). Seven out of 50 tests (14%) resulted in a change in cerebral hemodynamic targets.

**Conclusion:**

Findings from this series of children with TBI indicate that: (1) Availability of clinical order set with protocol facilitated clinical cerebral autoregulation testing, (2) Clinicians ordered cerebral autoregulation tests in children with severe TBI receiving high therapeutic intensity and repeatedly with impaired status on the first test, (3) Clinical cerebral autoregulation testing is feasible and safe, and (4) Testing results led to change in hemodynamic targets in some patients.

## Introduction

Traumatic Brain Injury (TBI) is a global public health burden. According to the Centers for Disease Control and Prevention, children (0–17 years) in the United States had 16,070 TBI-related hospitalizations in 2019 and 2,774 TBI-related deaths in 2020 (1). Research shows that children with complex mild TBI (2) and moderate-severe TBI (3) often have impaired cerebral autoregulation during the first week after TBI that resolves within a week in complex mild TBI (2) or persists for a more extended period after moderate-severe TBI (4). Impaired cerebral autoregulation is associated with worse clinical outcomes (3, 5, 6).

Cerebral autoregulation can be tested at the bedside using static or dynamic methods, depending on patient factors and expertise with methodologies. Transcranial Doppler (TCD) technology is often used to estimate changes in cerebral blood flow. Tilt testing methods recently documented intact cerebral autoregulation in healthy-term newborns (7). Most of our understanding of the prevalence, significance, and outcomes of impaired cerebral autoregulation in children is derived from clinical research studies (2, 8, 9). A review of our local IRB-approved research experiences testing cerebral autoregulation in over 96 children hospitalized with TBI over the past decade documented no adverse events. Given the potential clinical benefit of cerebral autoregulation testing, our interdisciplinary team (authors MSV, AM, RMC, MAK) collaborated to introduce an order set with testing protocol in the pediatric intensive care unit. We noted that clinicians at our facility use testing results to guide clinical decision-making, including adjusting ICP and CPP targets and the therapeutic intensity level. We thought that the availability of an order set with testing protocol might facilitate using cerebral autoregulation testing and standardize the interpretation of results. There is sparse data published about TCD ultrasonography use in clinical TBI care, and a lack of information on the feasibility/safety/complications associated with conducting these tests or the utility of information obtained to guide critical care (10).

In 2018, we incorporated a clinical protocol into an order set for cerebral autoregulation testing. We did not specify indications or timing for ordering these tests. The purpose of this report is to describe: (1) The clinical demand for clinical cerebral autoregulation testing in children hospitalized in the intensive care unit with TBI after implementing the order set with protocol, (2) The clinical setting in which cerebral autoregulation testing was performed, and (3) The feasibility and safety of clinical cerebral autoregulation testing. Secondarily, we evaluated the medical record for documentation of specific indications and outcomes of testing.

## Methods

### Institutional review board review and approval

This study (STUDY00015248) was reviewed and approved on 03/21/2022 by the Institutional Review Board of the University of Washington with a waiver of consent.

### Study setting and participation

This study was conducted at Harborview Medical Center (HMC), a 413-bed, Level I adult and pediatric trauma center. Patients under 18 years admitted between January 1, 2014, and December 31, 2021, were included. Children with severe TBI, moderate TBI, and complex TBI with and without polytrauma are admitted to a dedicated pediatric critical care unit and managed by a multidisciplinary team comprising members of the pediatric critical care and neurological surgical services. Of note, care for children with moderate and severe TBI was standardized at Harborview *via* the multidisciplinary hospital-wide PEGASUS program during this study period (11).

### Performance of TCD-based cerebral autoregulation tests

Harborview Medical Center has a clinical cerebrovascular laboratory with certified vascular technologists who conduct cerebral autoregulation tests for clinical and research purposes under physician supervision and written protocols, which have been operationalized into order sets that can be activated by providers (https://www.uwmedicine.org/locations/cerebrovascular-lab-harborview). The HMC cerebrovascular laboratory has over 40 years of research and clinical experience with dynamic and static cerebral autoregulation testing in adults and children. The pediatric order set for cerebral autoregulation testing was developed and implemented in 2018 after several successful research studies were performed since 2014 and after evaluating local experience with testing protocols by authors (RMC, MSV, MAK, AM) for clinical guidance and research experience and expertise (RMC and MSV).

The electronic order set in the electronic medical record (Supplementary Digital Content S1) details clinical conditions under which static or dynamic cerebral autoregulation testing can be carried out and the triggers (hemodynamic instability or elevated ICP) that prompt the TCD technician to stop testing. The order set also contains triggers for bedside physician consultation before, during, and after testing. Activation of cerebral autoregulation testing must be by a physician, and the ordering physician must indicate the type of cerebral autoregulation testing (pharmacological vs. tilt vs. thigh cuff) stimulus. This decision is left to the discretion of the ordering clinician. Typically, orders are placed by pediatric intensivists or neurological surgeons (although the initial request to perform the autoregulation test is universally made by the neurological surgeons). A detailed report of the cerebral autoregulation test result is then entered into the electronic medical record. It includes baseline vital signs (blood pressure, ICP, CPP, end-tidal (et-), or partial pressure of carbon dioxide (PCO2) at the time of testing, as well as post-testing vital signs) (Supplementary Digital Content S1). The report also includes information on if a test is completed or aborted. If tests are aborted and testing is not completed, there is documentation of reasons for not completing testing and any complications arising during testing.

### Data collection

For this analysis, TCD data and cerebral autoregulation assessments were identified from the case logs maintained by the HMC cerebrovascular laboratory. We abstracted demographical data such as age, sex, and race, as well as admission diagnosis, abnormalities reported on the computerized tomography of the head (CT) obtained at the time of admission, admission Glasgow Coma Scale score (GCS), discharge GCS, intensive care unit, and hospital length of stay (LOS), and discharge disposition. Transcranial Doppler (TCD) autoregulation data included the day the first and the last assessment was performed, the time from admission to the day of the first and last TCD-autoregulation study (days), and the total number of assessments/patient (sum, median, and range). We examined data on vital signs recorded during the TCD-autoregulation study, such as SBP, MAP, ICP, CPP, HR, and et-CO2/PCO2. To understand the clinical context in which testing was carried out, we examined the testing environment with invasive hemodynamic and brain monitors, sedation, analgesia, and vasoactive medications administered at the time of testing. We reviewed the progress notes of the bedside nurse, pediatric critical care team, and the neurological surgical service for documentation of contemporaneous change/no change in ICP/CPP/MAP targets in relation to the results of the autoregulation testing.

### Outcomes

Primary outcomes were: (1) demand for cerebral autoregulation testing, defined as the use of cerebral autoregulation testing, (2) feasibility of use, defined as the percent completed cerebral autoregulation tests; and (3) safety, which was defined by the absence of high ICP, hemodynamic instability, or reductions in CPP observed during testing. We also described the clinical setting in which cerebral autoregulation testing was ordered and the clinical changes made because of the testing results.

### Cerebral autoregulation testing and determining autoregulatory index

The change in head and back position proceeded from supine to upright position (13.6 cm/10 mmHg difference between two positions) served as the stimulus for testing cerebral autoregulation (12). For the relatively upright position, the vertical distance between the non-invasive blood pressure cuff and the external auditory meatus was used to calculate the estimate mean arterial pressure (MAPe) at the Circle of Willis. Because the mean arterial pressure decreases by 1 mmHg for every 1.36 cm increase in vertical height, the change in height from supine to upright was divided by 1.36 to calculate the MAPe in the sitting position (13–15). “Target MAPe was a decrease in MAPe 10 mmHg between supine and tilt, which serves as the autoregulatory stimulus during testing.” Autoregulatory index (ARI) for each middle cerebral artery was calculated off-line. Mathematically, the cerebral autoregulation was quantified using the ARI, where ARI = %ΔeCVR/%ΔMAPe, where eCVR is the estimated cerebrovascular resistance calculated as the ratio of MAP to Vmca as appropriate. An ARI of 0 represents absent autoregulation (pressure-dependent Vmca), whereas an ARI of 1.0 represents perfect autoregulation. For the purpose of statistical analysis, we dichotomized results into intact and impaired cerebral autoregulation. Impaired cerebral autoregulation (main outcome) was defined as unilateral or bilateral ARI less than 0.4 (16).

Impaired cerebral autoregulation was defined as an autoregulatory index <0.4, with an ARI of 0 reflecting absent cerebral autoregulation and an ARI of 0.4–1.0 indicating intact cerebral autoregulation (16).

### Data analysis

Descriptive statistics detailed patient characteristics. Categorical data were expressed as counts and percentages. After testing for normality distribution using the Shapiro-Wilk test, continuous variables (age, GCS, ICU/hospital LOS) were expressed as median and interquartile ranges (Q3–Q1). Patients with admission GCS of 13–15 were categorized as mild TBI, GCS 9–12 were categorized as moderate TBI, and patients with GCS 3–8 were defined as severe TBI. Categorical variables were compared using the Chi-Square test, and relative risk (RR) and 95% confidence interval were calculated. A *p*-value <0.005 indicated statistical significance. STATA (17)/RStudio version 1.554 (18) was used for statistical analysis.

## Results

### Patient characteristics

As Table 1 shows, the final sample consisted of 25 children with TBI, 24 with severe TBI, and 1 with complex mild TBI (median age of 13 years [IQR 4.5; 15] and median admission GCS of 3[IQR 3; 3.5]). Patients were primarily male (*n* = 19, 76%), typically with motor vehicle collision (*n* = 17, 68%), and subdural hematoma (*n* = 15, 60%) on head CT scan. Polytrauma (observed in 20, 80%) included the following body organ systems: extremities: *n* = 10 (50%), thoracic: *n* = 7 (35%), and abdomen: *n* = 3 (15%). Patients were Caucasian: *n* = 15 (60%), Hispanic: *n* = 7 (28%), African American: *n* = 2 (8%), and Asian: *n* = 1 (4%). The median intensive unit length of stay was 23 days [IQR 3.2; 32], and the median hospital length of stay was 23 days [IQR 16.5; 32]. Most (*n* = 20, 80%) patients were discharged to a rehabilitation facility, and 3 (12%) died.

**Table 1:** Characteristics of hospitalized children with traumatic brain injury who underwent clinical cerebral autoregulation testing.

	Overall (*n* = 25)
**Age in years (median, interquartile range, IQR)**	13 [4.5;15]
**Male sex *n* (%)**	19 (76%)
**Caucasian race/ethnicity *n* (%)** *	15 (60%)
**Mechanism of injury**	
Motor vehicle collision	17 (68%)
Falls	7 (28%)
Penetrating injury	1 (4%)
**Polytrauma**	20 (80%)
**Admission Glasgow Coma Scale Score (median, IQR)**	3 [3;3.5]
**Abnormalities on admission computerized tomography of head *n* (%)**	
Subdural hematoma	15 (60%)
Skull fracture	13 (52%)
Subarachnoid hemorrhage	11 (44%)
Intraparenchymal hemorrhage	7 (28%)
Pneumocephalus	5 (20%)
Cerebral contusions	4 (16%)
**Intensive unit length of stay in days (median, IQR)**	23 [13;32]
**Hospital length of stay in days (median, IQR)**	23 [16.5;32]
**Tracheostomy *n* (%)**	3 (12%)
**Gastrostomy feeding tube *n* (%)**	6 (24%)
**Discharge Glasgow Coma Scale score median [IQR]**	13 [10;15]
**Discharge disposition *n* (%)**	
Transfer to a rehabilitation facility	20 (80%)
Expired	3 (12%)
Home	1 (4%)
Transfer to an acute care facility	1 (4%)

* See details in the results section.

### Clinical setting

Over 7 years, 61 studies (92% static testing, 8% tilt testing, 0% with dynamic thigh cuff pressure release, and 66% of studies with intravenous vasoactive agents) were conducted. Due to the high prevalence of extremity polytrauma dynamic thigh-cuff pressure release tests are not first choice since they can result in significant pain. Similarly, the hyperemic response tests are not favored due to concurrent cerebrovascular injury which poses risk for carotid compression-based testing. The majority of testing was performed during 2018–2021 [post-order set implementation, *n* = 16, 64% (2018–2021) vs. *n* = 9, 36% (2014–2017) before order set].

The median time from admission to the first cerebral autoregulation test was 2 days [IQR 1; 4], and the median time from admission to the last test was 3 days [IQR 1.5; 7]. One patient had repeated cerebral autoregulation assessments for 16 days post-admission. Table 2 highlights the clinical setting in which cerebral autoregulation testing was performed. Tests were ordered and conducted between 0 and 16 days.

**Table 2:** Clinical setting of transcranial Doppler ultrasonography based cerebral autoregulation testing was performed.

	Total tests (*n* = 61)
**Time from admission to the first study in days median [IQR]**	2 [1;4]
**Glasgow Coma Scale score median [IQR]**	6 [3;7]
**Cerebrovascular hemodynamic profile**	
Heart rate bpm, median [IQR]	87 [70;98]
Mean arterial pressure (mmHg) Median [IQR]	79 [73;87]
Cerebral perfusion pressure (mmHg) Median [IQR] (*n* = 53)	66 [58;78]
Partial pressure carbon dioxide (torr) Median [IQR] (*n* = 55)	37 [35;39]
Intracranial pressure (mmHg) Median [IQR] (*n* = 55)	13 [9.5;19.5]
**Mechanically ventilated**	60 (98.4%)
**Monitoring**	
Invasive arterial blood pressure	60 (98.4%)
Central venous access catheter	60 (98.4%)
Intracranial pressure	56 (90.3%)
Brain tissue oxygenation	56 (90.3%)
Electroencephalography	38 (61.3%)
External ventricular drain	13 (25.5%)
**Pharmacotherapy**	
Vasoactive medications	55 (90.2%)
Sedation and analgesia	52 (83.8%)
Hyperosmolar therapy (Hypertonic saline/mannitol)	11 (17.7%)
**Neurosurgical intervention performed before the autoregulation test**	
Decompressive craniectomy	8 (12.9%)
Craniotomy	6 (9.7%)

IQR, interquartile range.

Average time of cerebral autoregulation assessment, median GCS was 6 [IQR 3; 7]; majority were mechanically ventilated (*n* = 60, 98.4%) with an invasive arterial blood pressure monitor (*n* = 60, 98.4%), central venous access catheter (*n* = 60, 98.4%), intracranial pressure monitor (*n* = 56, 90.3%), brain-tissue oxygenation monitor (*n* = 56, 90.3%), and with an external ventricular drain (*n* = 13, 25.5%). Most patients received sedation and analgesia for intracranial pressure control (*n* = 52. 83.8%) and vasoactive support (*n* = 55, 90.2%) during cerebral autoregulation testing.

### Feasibility of use and safety

**Figure 1:**
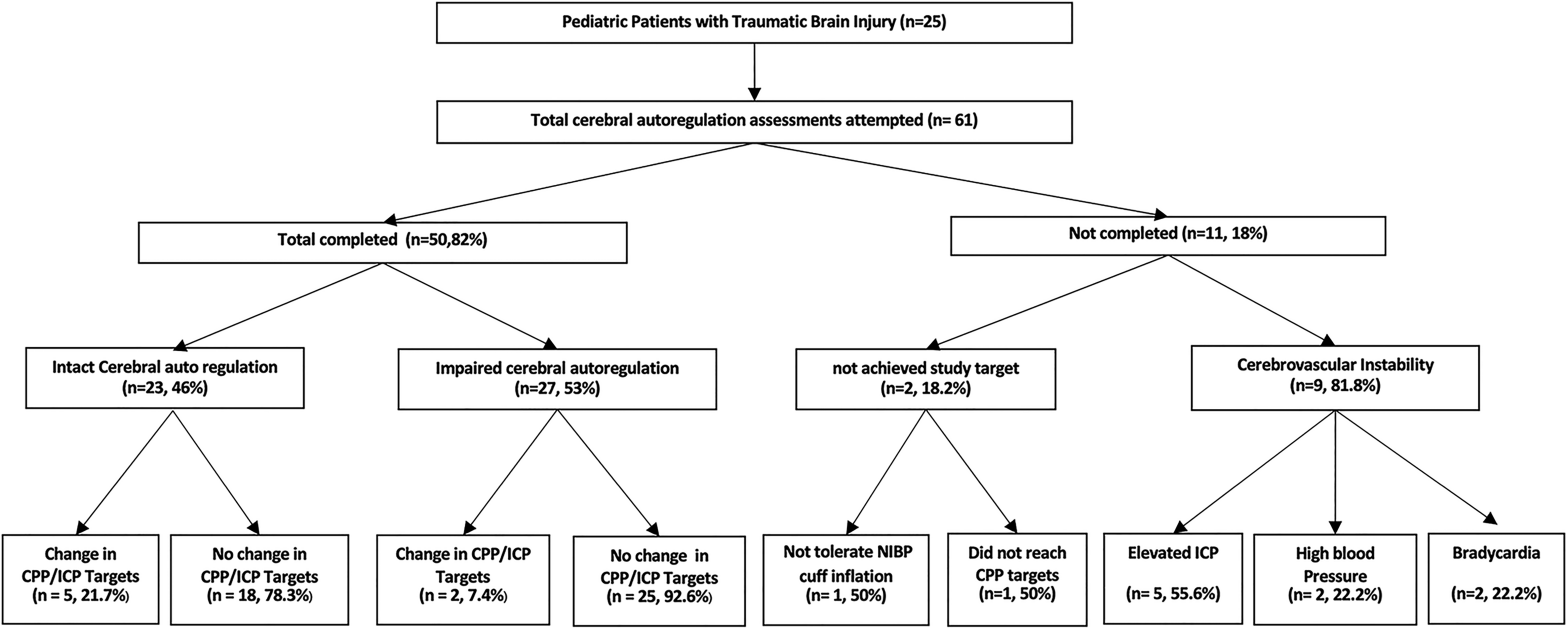
Performance of cerebral autoregulation assessments in hospitalized children with traumatic brain injury. ICP, intracranial pressure; CPP, cerebral perfusion pressure.

Cerebral autoregulation studies were completed in 50 (82%) attempted tests. Eleven tests were aborted due to elevated intracranial pressure (*n* = 5/9, 55.6%), reduction in cerebral perfusion pressure (*n* = 1/2, 50%), intolerance to blood pressure cuff (*n* = 1/2, 50%), bradycardia (*n* = 2/9, 22.2%), and high blood pressure (2/9, 22.2%) as shown in Figure 1.

### Cerebral autoregulation findings

Ten (47.6%) of the first assessments demonstrated impaired/absent cerebral autoregulation. Abnormal/absent cerebral autoregulation on the first cerebral autoregulation assessment was associated with repeat cerebral autoregulation testing (80% vs. 27.3%, RR 2.93, 95% CI 1.06:8.08, *p* = 0.03).

### Utility of cerebral autoregulation test results

Changes in intracranial pressure and/or cerebral perfusion pressure targets were made due to data obtained from 7 out of 50 completed tests (14%). Impaired/absent cerebral autoregulation was associated with changes in intracranial pressure targets after two (7.4%) tests. Intact cerebral autoregulation documented changes in intracranial pressure (4/23, 17.4%), cerebral perfusion pressure (1/23, 4.3%), and mean arterial pressure (0/23, 0%) targets. In most clinical settings, cerebral hemodynamic targets were changed, and elevated intracranial pressure was treated with hyperosmolar agents. After changing cerebral hemodynamic targets, the result was a higher tolerated intracranial pressure (ICP target of 25 mmHg instead of 22 mmHg).

## Discussion

In this study, we examined our experience with clinical cerebral autoregulation testing in children hospitalized with TBI after implementing a cerebral autoregulation testing order set with a protocol. The main findings are that clinical cerebral autoregulation tests were: (1) availability of clinical order set with protocol facilitated clinical cerebral autoregulation testing, (2) clinicians ordered cerebral autoregulation tests in children with severe TBI receiving high therapeutic intensity and repeatedly with impaired status on the first test, (3) clinical cerebral autoregulation testing is feasible and safe, and (4) testing results may be associated with a change in hemodynamic targets in some patients. To our knowledge, this is the first report on the safety and feasibility of the use of TCD-based cerebral autoregulation status in pediatric TBI for clinical use.

Our review shows that clinicians ordered cerebral autoregulation tests more after order set implementation. Despite decades of institutional TCD-based cerebral autoregulation testing experience, overall clinical use of cerebral autoregulation testing was lower than when formally acknowledged as a test with potential clinical utility by having a formal order set with a protocol. Our past research helped us propel the translation of cerebral autoregulation testing technology into acceptance of this test for use in clinical practice. What was also helpful was the co-location and lessons learned from adult practice; given that Harborview Medical Center is a mixed level 1 adult and pediatric trauma center as well as a center of neuroscience excellence where TCD technology is commonly used to assess the cerebral autoregulatory status and diagnose cerebrovascular conditions such as vasospasm after subarachnoid hemorrhage. We think that lessons learned from the adult side helped facilitate the translation of pediatric TBI research to pediatric TBI care and from adult neurocritical care to pediatric TBI care. Results of this study show that the availability of an order set with a protocol that legitimizes cerebral autoregulation testing in clinical use led to higher clinician utilization.

We learned from this study that clinicians ordered cerebral autoregulation tests in children with TBI and high therapeutic intensity and repeatedly in those with initially abnormal test results. Research shows that patients with severe TBI may have impaired cerebral autoregulation (19, 20), associated with severe TBI category (21), which may increase the risk for cerebral hypoperfusion. Systemic hypotension causes cerebral vasodilation, increases cerebral blood volume, and drives intracranial hypertension, which may further impair cerebral autoregulation. Patients with severe TBI require mechanical ventilation (22), and sedative agents such as propofol are commonly administered to allow ventilator tolerance, ICP control, and reduced cerebral metabolic demand for oxygen (23, 24). Vasoactive medications (typically norepinephrine in this patient population) allow for the maintenance of systemic and cerebral perfusion pressure targets (25). These data show clinicians ordered cerebral autoregulation testing in children with the most severe TBI (GCS 3–7). Data supporting this conjecture includes the high percentage of patients with significant CT lesions, high rates of gastrostomy tube placement, tracheostomy, LOS longer than a week, and high rate of discharge to inpatient rehabilitation. Other reasons why clinicians may have ordered cerebral autoregulation tests include prognostication, investigating the safety of adjusting systemic and cerebral hemodynamic goals (systolic blood pressure, mean arterial pressure, cerebral perfusion pressure, and ICP), as well as deliberate clearance for surgery or diagnosis and differentiate between cerebral hyperemia vs. cerebral vasospasm. These options and our data suggest that clinicians will use the results of cerebral autoregulation testing to individualize TBI care in some patients.

We examined the feasibility of testing completion and the safety of cerebral autoregulation testing. Our data suggest that TCD autoregulation assessments are feasible and safe with a low complication rate. In all cases, the order set provided parameters for conducting these tests with thresholds for cessation of testing. Unlike prior research studies, there was no requirement for a physician to be at the patient’s bedside, but that one should be available should there be a complication. Our data suggest that this proviso is acceptable for such a protocol. However, it is essential to note that cerebral autoregulation testing, as we have conducted, intervenes and alters physiology; it is crucial to pre-specify, understand, note, and address changes in ICP, reductions in CPP, systemic hypertension, or bradycardia, which may prompt testing to be stopped. We think an explicit mention of potential complications associated with testing during static and dynamic TCD-based cerebral autoregulation testing and situational awareness from the critical care providers allow for safe testing. Reporting complications to an institutional quality improvement database with a periodic discussion of these events may promote a safety culture around static and dynamic cerebral autoregulation testing. We anticipated clinicians might order cerebral autoregulation testing and use this information to guide prognosis, optimize systemic and cerebral hemodynamic targets and the timing of surgery, and better understand TBI evolution at the patient level.

It has been over 50 years since Lassen first proposed the concept of autoregulation (26). Research has taken significantly longer than the suggested 17-year time lag to reach clinical practice (27). While safety considerations may have contributed to reluctance in ordering autoregulation testing in children with less severe TBI, changes in clinical practice take time. Translating basic science research to patient benefit and clinical practice is a process that involves learning new and unlearning older modalities of care (28). A modified Becker model for unlearning in medicine (from the business world) characterizes the process of physician unlearning as a product of the tension between prior knowledge influenced by—a prior mental model, characteristics of the physician, physicians’ beliefs about consequences to patients, social influences—and new knowledge (28, 29). Results of a qualitative study to understand the experience of clinical practice change in primary practice physicians identified physician characteristics like personal bias, clinical experiences, and openness to change as sources of tension for unlearning (28). Physicians reported that the opinions of different physicians and discussions on how to incorporate data into practice were needed in addition to the literature to change their practice. The clinical practice gradually builds comfort in a dynamic rather than a fixed unidirectional process.

This study has some strengths and limitations. The main strength is that we detail the rationale, process, and outcomes of implementing a novel clinical order set with cerebral autoregulation testing protocol that results from translating research to practice. Our experience also allows us to advance the practice of advanced neuromonitoring in pediatric TBI care. Limitations are the small sample size and a single-center study and that indications for testing were not defined. We cannot generalize feasibility and safety data to other institutions, which may differ at institutions with varying characteristics. TCD laboratory structure, expertise, and experience may have different results with clinical use and testing outcomes. The utility of clinical cerebral autoregulation testing may vary in mild-moderate TBI or patients with TBI with different injury characteristics.

## Conclusion

Findings from this series of children with TBI indicate that: (1) Availability of a clinical order set with protocol facilitated clinical cerebral autoregulation testing, (2) Clinicians ordered cerebral autoregulation tests in children with severe TBI receiving high therapeutic intensity and repeatedly with impaired status on the first test, (3) Clinical cerebral autoregulation testing is feasible and safe, and (4) Testing results led to change in hemodynamic targets in some patients.

## Data Availability

The raw data supporting the conclusions of this article will be made available by the authors, without undue reservation.
