# Supplementary material for: Experience with clinical cerebral autoregulation testing in children hospitalized with traumatic brain injury: Translating research to bedside
Source: Front Pediatr. 2023 Jan 10;10:1072851. doi: 10.3389/fped.2022.1072851 (PMC9871541; doi:10.3389/fped.2022.1072851)
Supplement: Supplementary file 1 [file Datasheet1.pdf]

Supplementary Digital Content 1

Transcranial Doppler Cerebral Autoregulation Testing Order Set and Testing report

Order Set Name: Autoregulation testing

- **Vital Signs**
- **End-tidal carbon dioxide (CO2) monitoring**
- **Vasopressors**
  - **After baseline TCD:**
    - If the patient is already on vasopressor, titrate to the maximum dose allowed to achieve the cerebral perfusion pressure goal.
    - Once testing is complete, wean vasopressor to pre-testing dose to meet desired parameters or to wean off
      - **Choice of agents:**
        - Phenylephrine
        - Norepinephrine
- **Arterial blood gas: perform just prior to the test, note end-tidal CO2 at blood draw, and correlate**
- **Type of autoregulation test**
  - **Static: Vascular ultrasound autoregulation test:**
    - Record baseline MAP, ICP, and CPP. Once CPP 20 is above baseline, record MAP, ICP, CPP, end-tidal CO2
  - **Tilt: Vascular ultrasound autoregulation test:** Record baseline MAPs supine and sitting

Autoregulation testing report
Study date and time _

Physiological Findings:
Heart Rate: _ bpm
Hematocrit: _ %
Serum sodium: _mEq/L
Glasgow Coma Scale score: _

Autoregulation Studies:
Study type: Static Autoregulation
Baseline:
MAP: _ mmHg, RMCA: _ cm/s, LMCA: _ cm/s, ICP: _ mmHg, CPP: _ mmHg.
EtCO2: _ from arterial blood gas
Target:
MAP: _ mmHg, RMCA: _ cm/s, LMCA: _ cm/s, ICP: _ mmHg, CPP: _ mmHg.
Post Vasopressor:
MAP: _ mmHg, RMCA: _ cm/s, LMCA: _ cm/s, ICP: _ mmHg, CPP: _ mmHg.
Left MCA Autoregulation Index: _
Right MCA Autoregulation Index: _

Study status: Complete/Incomplete
Complications: _
Reasons for incomplete study: _

Preliminary Findings:
Static Autoregulation demonstrates:

**1.** Intact/impaired cerebral autoregulation right MCA
2. Intact/impaired cerebral autoregulation left MCA

Abbreviations: *MAP: mean arterial blood pressure; ICP: intracranial pressure; CPP: cerebral perfusion pressure; MCA: Middle cerebral artery, L: Left-sided. R: right-sided*
